# Analyzing Three-Player Quantum Games in an EPR Type Setup

**DOI:** 10.1371/journal.pone.0021623

**Published:** 2011-07-27

**Authors:** James M. Chappell, Azhar Iqbal, Derek Abbott

**Affiliations:** 1 School of Chemistry and Physics, University of Adelaide, Adelaide, South Australia, Australia; 2 School of Electrical and Electronic Engineering, University of Adelaide, Adelaide, South Australia, Australia; Hungarian Academy of Sciences, Hungary

## Abstract

We use the formalism of Clifford Geometric Algebra (GA) to develop an analysis of quantum versions of three-player non-cooperative games. The quantum games we explore are played in an Einstein-Podolsky-Rosen (EPR) type setting. In this setting, the players' strategy sets remain identical to the ones in the mixed-strategy version of the classical game that is obtained as a proper subset of the corresponding quantum game. Using GA we investigate the outcome of a realization of the game by players sharing GHZ state, W state, and a mixture of GHZ and W states. As a specific example, we study the game of three-player Prisoners' Dilemma.

## Introduction

The field of game theory [1], [2] has a long history [3], but was first formalized in 1944 with the work of von Neumann and Morgenstern [4], aiming to develop rational analysis of situations that involve strategic interdependence.

Classical game theory has found increasing expression in the field of physics [3] and its extension to the quantum regime [5] was proposed by Meyer [6] and Eisert et al [7], though its origins can be traced to earlier works [8]–[11]. Early studies in the area of quantum games focused on the two-player two-strategy non-cooperative games, with the proposal for a quantum Prisoners' Dilemma (PD) being well known [7]. A natural further development of this work was its extension to multiplayer quantum games that was explored by Benjamin and Hayden [12]. Du et al. [13], [14] explored the phase transitions in quantum games for the first time that are central in the present article.

The usual approach in three-player quantum games considers players sharing a three-qubit quantum state with each player accessing their respective qubit in order to perform local unitary transformation. Quantum games have been reported [15] in which players share Greenberger-Horne-Zeilinger (GHZ) states and the W states [5], while other works have, for instance, investigated the effects of noise [16], [17] and the benefits of players forming coalitions [18], [19].

A suggested approach [20]–[23] in constructing quantum games uses an Einstein-Podolsky-Rosen (EPR) type setting [24]–[31]. In this approach, quantum games are setup with an EPR type apparatus, with the players' strategies being local actions related to their qubit, consisting of a linear combination (with real coefficients) of (spin or polarization) measurements performed in two selected directions.

Note that in a standard arrangement for playing a mixed-strategy game, players are faced with the identical situation, in that in each run, a player has to choose one out of two pure strategies. As the players' strategy sets remain classical, the EPR type setting avoids a well known criticism [32] of quantum games. This criticism refers to quantization procedures in which players are given access to extended strategy sets, relative to what they are allowed to have in the classical game. Quantum games constructed with an EPR type setting have been studied in situations involving two players [22] and also three players [23]. The applications of three-player quantum games include describing three-party situations, involving strategic interaction in quantum communication [33].

In recent works, the formalism of Clifford's geometric algebra (GA) [34]–[38] has been applied to the analysis of two-player quantum games with significant benefits [39], [40], and so is also adopted here in the analysis of three-player quantum games. The use of GA is justified on the grounds that the Pauli spin algebra is a matrix representation of Clifford's geometric algebra in 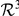, and hence we are choosing to work directly with the underlying Clifford algebra. There are also several other documented benefits of GA such as:

- The unification of the dot and cross product into a single product, has the significant advantage of possessing an inverse. This results in increased mathematical compactness, thereby aiding physical intuition and insight [41].
- The use of the Pauli and Dirac matrices also unnecessarily introduces the imaginary scalars, in contrast to GA, which uses exclusively real elements [42]. This fact was also pointed out by Sommerfield in 1931, who commented that ‘*Dirac's use of matrices simply rediscovered Clifford algebra*’ [43].
- In the density matrix formalism of quantum mechanics, the expectation for an operator 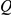 is given by 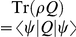, from which we find the isomorphism to GA, Tr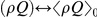, the subscript zero, indicating to take the scalar part of the algebraic product 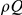, where 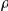 and 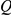 are now constructed from real Clifford elements. This leads to a uniquely compact expression for the overlap probability between two states in the 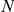-particle case, given by Eq. (13), which allows straightforward calculations that normally require 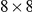 complex matrices representing operations on three qubits.
- Pauli wave functions are isomorphic to the quaternions, and hence represent rotations of particle states [44]. This fact paves the way to describe general unitary transformations on qubits, in a simplified algebraic form, as *rotors*. In regard to Hestenes' analysis of the Dirac equation using GA, Boudet [41] notes that, ‘the use of the pure real formalism of Hestenes brings noticeable simplifications and above all the entire geometrical clarification of the theory of the electron. ’
- Recent works [6], [39], [40] show that GA provides a better intuitive understanding of Meyer's quantum penny flip game [6], using operations in 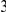-space with *real coordinates*, permitting helpful visualizations in determining the quantum player's winning strategy. Also, Christian [45], [46] has recently used GA to produce thought provoking investigations into some of the foundational questions in quantum mechanics.

Our quantum games use an EPR type setting and players have access to general pure quantum states. We determine constraints that ensure a faithful embedding of the mixed-strategy version of the original classical game within the corresponding quantum game. We find how a Pareto-optimal quantum outcome emerges in three-player quantum PD game at high entanglement. We also report phase transitions taking place with increasing entanglement when players share a mixture of GHZ and W type states in superposition.

In an earlier paper [23], two of the three authors contributed to developing an entirely probabilistic framework for the analysis of three-player quantum games that are also played using an EPR type setting, whereas the present paper, though using an EPR type setting, provides an analysis from the perspective of quantum mechanics, with the mathematical formalism of GA. The previous work analyzed quantum games from the non-factorizable property of a joint probability distribution relevant to a physical system that the players shared in order to implement the game. For the game of three-player Prisoners' Dilemma, our probabilistic analysis showed that non-factorizability of a joint probability distribution indeed can lead to a new equilibrium in the game. The three-player quantum Prisoners' Dilemma, in the present analysis, however, moves to the next step and explores the phase structure relating players' payoffs with shared entanglement and also the impact of players sharing GHZ and W states and their mixture. We believe that without using the powerful formalism of GA, a similar analysis will nearly be impossible to perform using an entirely probabilistic approach as developed in [22].

### EPR setting for playing quantum games

The EPR setting [20], [22], [23] two player quantum games involves a large number of runs when, in a run, two halves of an EPR pair originate from the same source and move in the opposite directions. Player Alice receives one half whereas player Bob receives the other half. To keep the non-cooperative feature of the game, it is assumed that players Alice and Bob are located at some distance from each other and are not unable to communicate between themselves. The players, however, can communicate about their actions, which they perform on their received halves, to a *referee* who organizes the game and ensures that the rules of the game are followed. The referee makes available two directions to each player. In a run, each player has to choose one of two available directions. The referee rotates Stern-Gerlach type detectors [5] along the two chosen directions and performs quantum measurement. The outcome of the quantum measurement, on Alice's side, and on Bob's side of the Stern-Gerlach detectors, is either 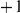 or 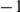. Runs are repeated as the players receive a large number of halves in pairs, when each pair comes from the same source and the measurement outcomes are recorded for all runs. A player's strategy, defined over a large number of runs, is a linear combination (with normalized and real coefficients) of the two directions along which the measurement is performed. The referee makes public the payoff relations at the start of the game and announces rewards to the players after the completion of runs. The payoff relations are constructed in view of a) the matrix of the game, b) the list of players' choices of directions over a large number of runs, and c) the list of measurement outcomes that the referee prepares using his/her Stern-Gerlach apparatus.

For a three-player quantum game, this setting is extended to consider three players Alice, Bob and Chris who are located at the three arms of an EPR system [5]. In the following they will be denoted by 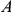, 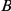 and 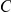, respectively. As it is the case with two-player EPR setting, in a run of the experiment, each player chooses one out of two directions.

We have used the EPR setting in view of the well known Enk and Pike's criticism [32] of quantum games that are played using Eisert et al's setting [7]. Essentially this criticism attempts to equate a quantum game to a classical game in which the players are given access to an extended set of classical strategies. The present paper uses an EPR setting in which each player has two classical strategies consisting of the two choices he/she can make between two directions along which a quantum measurement can be performed. That is, the player's pure strategy, in a run, consists of choosing one direction out of the two. As the sets of strategies remain exactly identical in both the classical and the quantum forms of the game, it is difficult to construct an Enk and Pike type argument for a quantum game that is played with an EPR setting.

**Figure 1:**
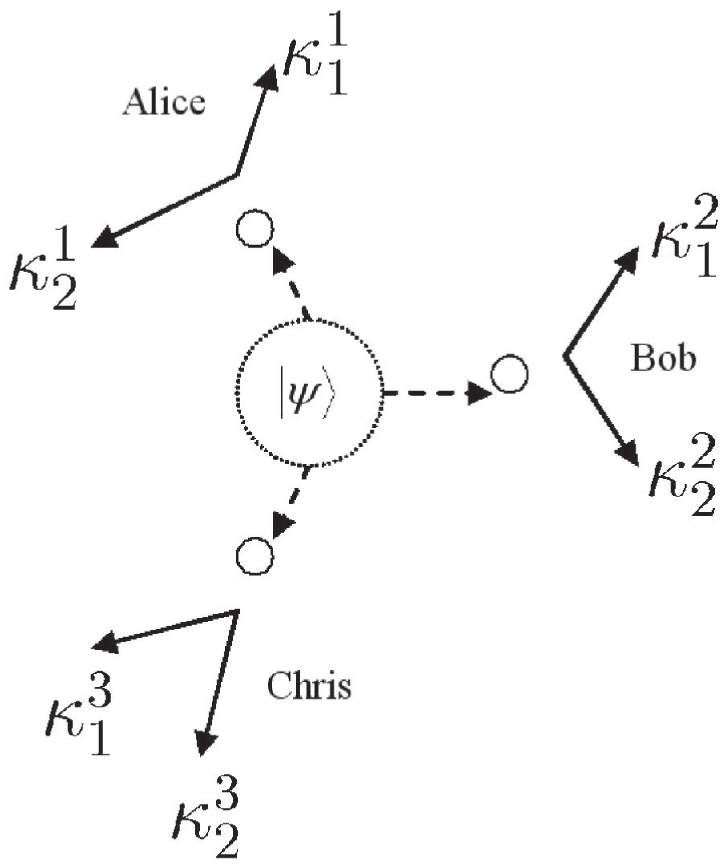
The EPR setup for three-player quantum game. A three-qubit entangled quantum state is distributed to the three players, who each choose between two possible measurement directions.

As Fig. 1 shows, we represent Alice's two directions as 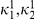. Similarly, Bob's directions are 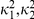 and Chris' are 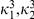. The players measurement directions form a triplet out of eight possible cases 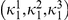, 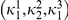, 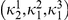, 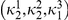, 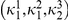, 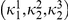, 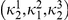, 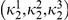 and measurement is performed along the chosen directional triplet. The measurement outcome for each player along their chosen direction is 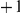 or 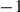.

Over a large number of runs the players sequentially receive three-particle systems emitted from a source and a record is maintained of the players' choices of directions over all runs. One of the eight possible outcomes 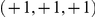, 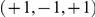, 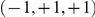, 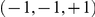, 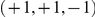, 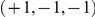, 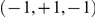, 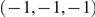 emerges out of the measurement in an individual run, with the first entry for Alice's outcome, the second entry for Bob's outcome and the third entry for Chris' outcome.

In the following we express the players' payoff relations in terms of the outcomes of these measurements. These payoffs depend on the triplets of the players' strategic choices made over a large number of runs and on the dichotomic outcomes of the measurements performed along those directions.

### Players' sharing a symmetric initial state

We consider the situation in which an initial quantum state of three qubits is shared among three players. To obtain a fair game, we assume this state is symmetric with regard to the interchange of the three players. The GHZ state is a natural candidate given by
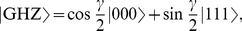
(1)where we have an entanglement angle 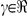, which has been shown [5] to be capable of producing the maximally entangled three qubit state. Alternatively we could start with the W entangled state
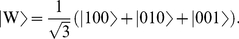
(2)The other symmetric state would be an inverted W state
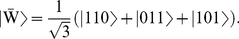
(3)


After the measurement along three directions selected by the players, each player is rewarded according to a payoff matrix 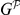, for each player 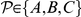. Thus the expected payoffs for a player is given by
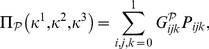
(4)where 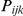 is the probability the state 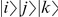 is obtained after measurement, with 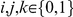, along the three directions 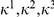 chosen by Alice, Bob and Chris respectively. In the EPR setting, 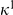 can be either of Alice's two directions i.e. 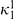 or 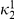 and similarly for Bob and Chris.

### Clifford's geometric algebra

The formalism of GA [34]–[38] has been shown to provide an equivalent description to the conventional tensor product formalism of quantum mechanics.

To set up the GA framework for representing quantum states, we begin by defining 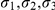 as a right-handed set of orthonormal basis vectors, with
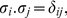
(5)where 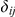 is Kronecker delta. Multiplication between algebraic elements is defined to be the geometric product, which for two vectors 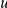 and 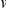 is given by
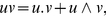
(6)where 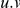 is the conventional symmetric dot product and 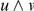 is the anti-symmetric outer product related to the Gibb's cross product by 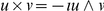, where 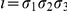. For distinct basis vectors we find
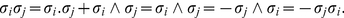
(7)This can be summarized by
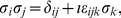
(8)where 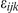 is the Levi-Civita symbol. We can therefore see that 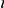 squares to minus one, that is 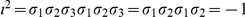 and commutes with all other elements and so has identical properties to the unit imaginary 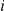. Thus we have an isomorphism between the basis vectors 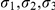 and the Pauli matrices through the use of the geometric product.

In order to express quantum states in GA we use the one-to-one mapping [36], [38] defined as follows
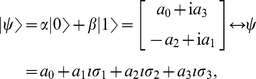
(9)where 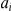 are real scalars.

For a single particle we then have the basis vectors
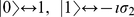
(10)and so for three particles we can use as a basis
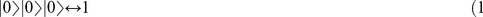
(11a)

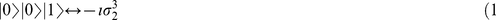
(11b)

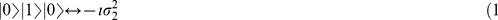
(11c)

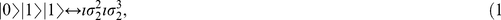
(11d)

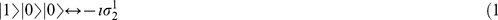
(11e)

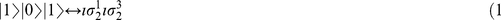
(11f)

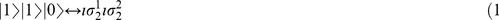
(11g)

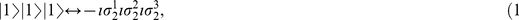
(11h)where to reduce the number of superscripts representing particle number we write 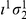 as 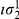. General unitary operations are equivalent to rotors in GA [36], represented as
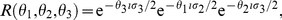
(12)which is in Euler angle form and can completely explore the available space of a single qubit. Using the definition of unitary operations given by Eq. (12) we define 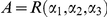, 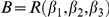, 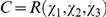 for general unitary transformations acting locally on each of the three players qubit in order to generalize the starting state, that is the GHZ or W states, as far as possible.

We define a separable state 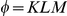, where 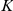, 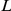 and 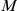 are single particle rotors, which allow the players' measurement directions to be specified on the first, second and third qubit respectively. The state to be measured is now projected onto this separable state 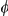. The overlap probability between two states 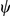 and 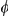 in the 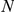-particle case is given in Ref. [36] as
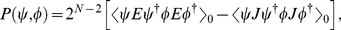
(13)where the angle brackets 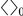 mean to retain only the scalar part of the expression and 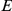 and 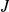 are defined for 3 particles in Ref. [36] as
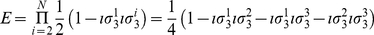
(14a)

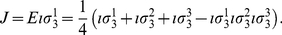
(14b)


The 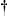 operator acts the same as complex conjugation: flipping the sign of 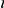 and inverting the order of the terms.

## Results

We now, firstly, calculate the observables from Eq. (11) for the GHZ state in GA, which from Eq. (11) gives
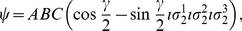
(15)where 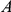, 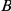, and 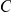 represent the referee's local unitary actions, written as rotors 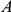, 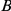, and 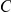 in GA, on the respective player's qubits, in order to generalize the starting state. Referring to Eq. (13), we firstly calculate
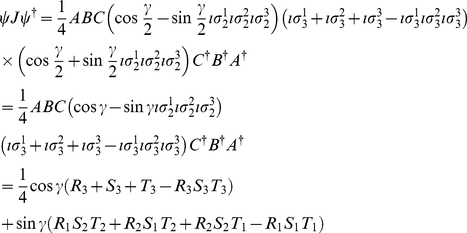
(16)where 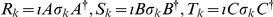. We also calculate
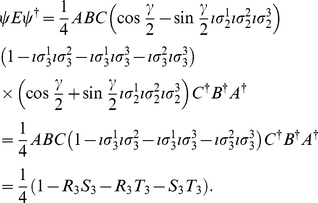
(17)For measurement defined with 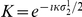, 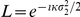 and 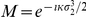 allowing a rotation of the detectors by an angle 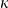, where we have written 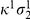 as 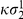, we find
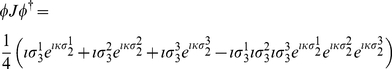
(18a)

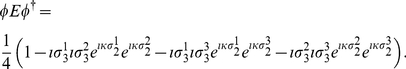
(18b)From Eq. (13) we find
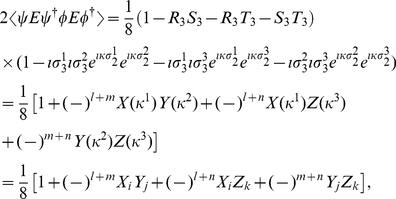
(19)where 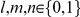 refers to measuring a 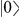 or 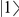 state, respectively, and using the standard results listed in the Appendix S1, we have
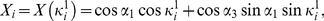
(20a)

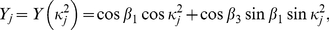
(20b)

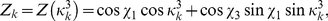
(20c)with 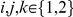, representing the two measurement directions available to each player. Also from Eq. (13) we have
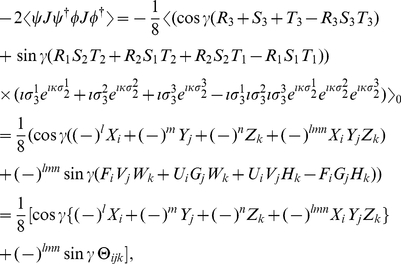
(21)where
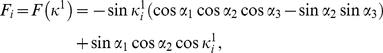
(22a)

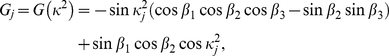
(22b)

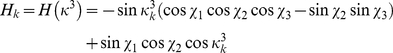
(22c)and
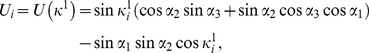
(23a)

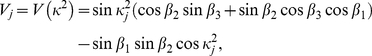
(23b)

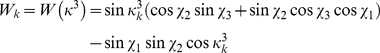
(23c)and
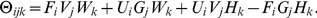
(24)So we find from Eq. (13) the probability to observe a particular state after measurement as
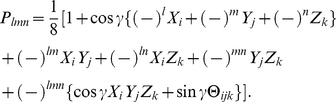
(25)


For instance, at 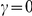 we obtain
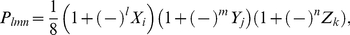
(26)which shows a product state, as expected. Alternatively with general entanglement, but no operation on the third qubit, that is 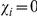, we have
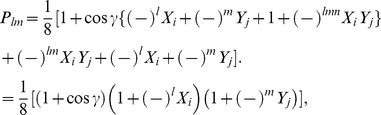
(27)which shows that for the GHZ type entanglement each pair of qubits is mutually unentangled.

### Obtaining the payoff relations

We extend the approach of Ichikawa and Tsutsui [47] to three qubits and represent the permutation of signs introduced by the measurement process. For Alice we define
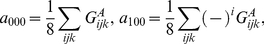
(28a)

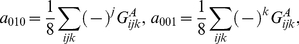
(28b)

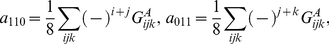
(28c)

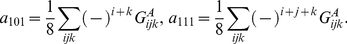
(28d)Using Eq. (4), we then can find the payoff for each player
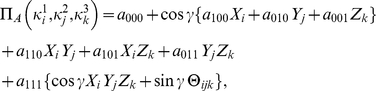
(29a)

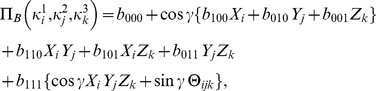
(29b)

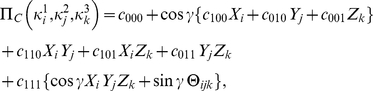
(29c)where, as Eqs. (20) show, the three measurement directions 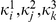 are held in 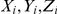. Alternatively, in order to produce other quantum game frameworks [7], [48], we can interpret the rotors 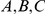, held in 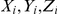, as the unitary operations which can be applied by each player to their qubit, where in this case, the measurement directions will be set by the referee.

#### Mixed-strategy payoff relations

For a mixed strategy game, Alice, Bob and Chris choose their first measurement directions 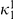, 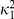, 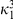 with probabilities 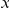, 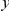 and 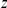 respectively, where 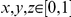 and hence choose the directions 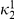, 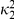, 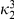 with probabilities 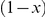, 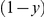, 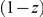, respectively. Alice's payoff is now given as
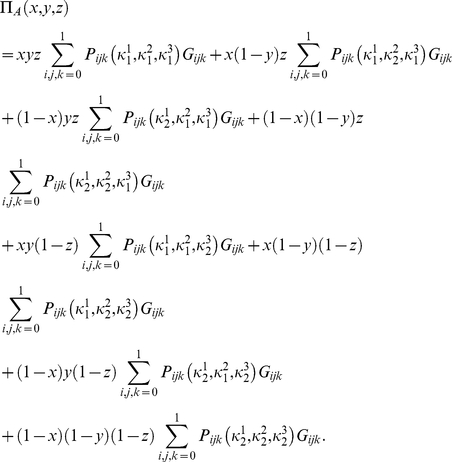
(30)


#### Payoff relations for a symmetric game

For a symmetric game we have 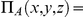 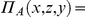 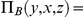 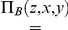 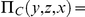 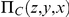. This requires 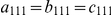, 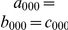, 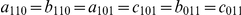, 
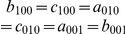
, 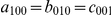 and 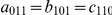. The payoff relations (0) are then reduced to
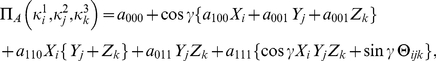
(31a)

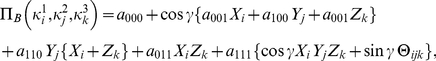
(31b)

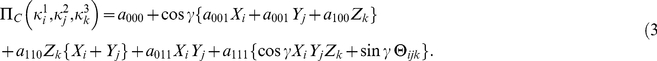
(31c)


### Embedding the classical game

If we consider a strategy triplet 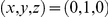 for example, at zero entanglement, then the payoff to Alice is obtained from Eq. (30) to be
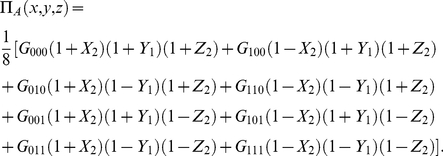
(32)Hence, in order to achieve the classical payoff of 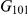 for this triplet, we can see that we require 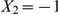, 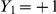 and 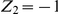.

This shows that we can select any required classical payoff by the appropriate selection of 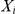, 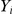, 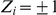. Referring to Eq. (20), we therefore have the conditions for obtaining classical mixed-strategy payoff relations as
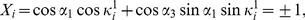
(33a)

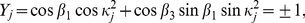
(33b)

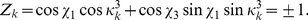
(33c)


For the equation for Alice, we have two classes of solution: If 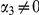, then for the equations satisfying 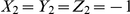 we have for Alice in the first equation 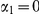, 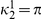 or 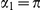, 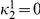 and for the equations satisfy 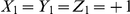 we have 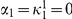 or 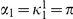, which can be combined to give either 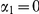, 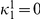 and 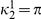 or 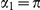, 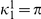 and 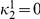. For the second class with 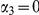 we have the solution 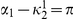 and for 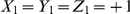 we have 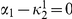.

So in summary for both cases we have that the two measurement directions are 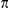 out of phase with each other, and for the first case (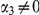) we can freely vary 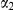 and 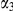, and for the second case (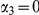), we can freely vary 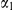 and 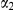 to change the initial quantum quantum state without affecting the game Nash equilibrium (NE) or payoffs [1], [2]. The same arguments hold for the equations for 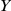 and 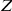. Using these results in Eq. (24) we find that 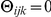.

We have the associated payoff for Alice
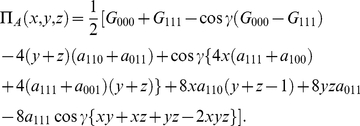
(34)Setting 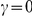 in Eq. (34) we find Alice's payoff as
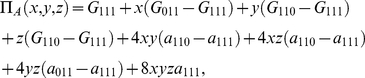
(35)which has the same payoff structure as the mixed-strategy version of the classical game.

Now, we can also write the equations governing the NE as
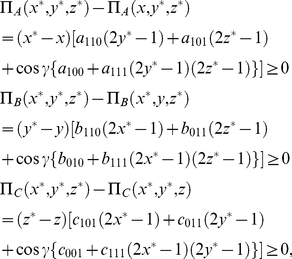
(36)where the strategy triple 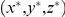 is a NE. Using the conditions defined earlier for a symmetric game, we can reduce our equations governing the NE for the three players to
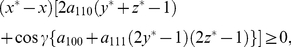
(37a)

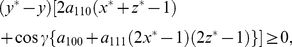
(37b)

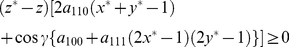
(37c)


We can see that the new quantum behavior is governed solely by the payoff matrix through 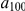, 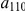 and 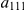 and by the entanglement angle 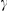, and not by other properties of the quantum state.

For completeness, we have Bob's payoff, in the symmetric case, as
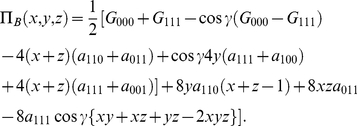
(38)


The mixed NE for all players is
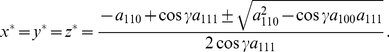
(39)


#### Maximally entangled case

For 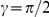 at maximum entanglement for both NE of 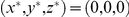 and 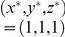 we have the payoff
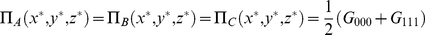
(40)which gives the average of the two corners of the payoff matrix, which is as expected.

#### Prisoners' Dilemma

An example of a three-player PD game is shown in Table 1. For this game, from Eq. (28), we have 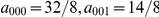, 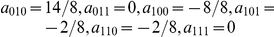, with the NE from Eqs. (37) given by
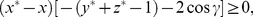
(41a)

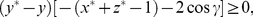
(41b)

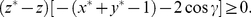
(41c)


**Table 1:** An example of three-player Prisoners' Dilemma.

State	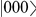	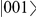	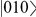	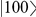	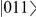	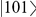	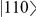	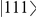
Payoff	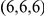	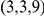	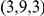	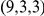	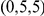	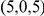	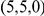	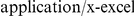

The payoff for each player (one,two,three), for each measurement outcome.

We have the classical NE of 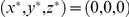 for 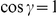, but we have a phase transition, as the entanglement increases, at 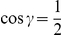 where we find the new NE 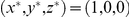, 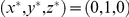 and 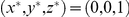. The payoff for Alice from Eq. (34) is given by
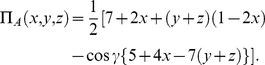
(42)


**Figure 2:**
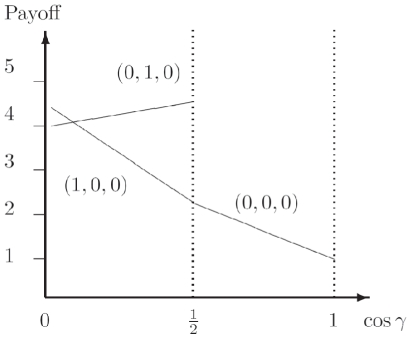
Phase structure for Alice in quantum PD game using EPR setting. For the PD example given in Table 1, the classical outcome of (0,0,0), is still returned for low entanglement, 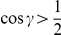, but with new NE arising at higher entanglement. As the game is symmetric, we have 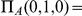 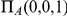 and the NE 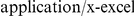 is not shown.

For the classical region we have 
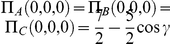
, which is graphed in Fig. 2 along with other parts of the phase diagram. It should be noted that 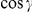 can go negative, which will produce a mirror image about the vertical axis of the current graph. That is for 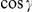 decreasing from 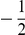 to 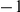, we have a NE of 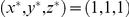, falling from 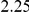 down to 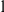. We will also have the NE of 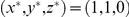 and 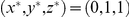 for 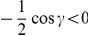.

This graph also illustrates the value of coalitions, because if Bob and Chris both agree to implement the same strategy, then the only NE available for 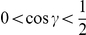 for example, is 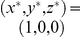. However, for a NE in the region of 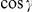 just less than one half, both Bob and Chris receive a significantly greater payoff, of around 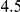 units, as opposed to 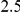 for Alice, so the coalition will receive nearly twice the payoff.

### Players sharing the W state

The second type of three particle entangled state [49] is the W state
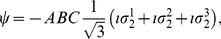
(43)where once again we have used the three rotors 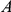, 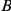 and 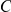 in order to generalize the state as far as possible. So proceeding as for the GHZ state, the probability that a particular state will be observed after measurement can be found to be
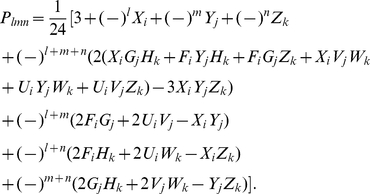
(44)Clearly the same probability distribution would be found for the second type of W state, shown in Eq. (3), because it is simply an inverse of this state.

#### Obtaining the pure-strategy payoff relations

With players sharing a W state, referring to Eq. (28), we introduce the following notation for Alice
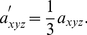
(45)Using the payoff function given by Eq. (4), we then find for Alice
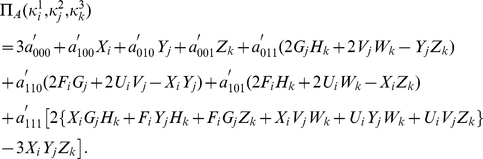
(46)Similarly for other players, simply by switching to their payoff matrix in place of Alices'.

Obviously for the W state there is no way to turn off the entanglement and so it is not possible to embed a classical game, hence we now turn to a more general state which is in a superposition of the GHZ and W type states.

### Games with general three-qubit state

It is noted in Ref. [49] that there are two inequivalent classes of tripartite entanglement, represented by the GHZ and W states. More specifically, Ref. [50] finds a general three qubit pure state
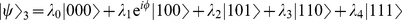
(47)where 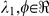, with 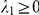, 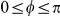 and 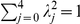.

We have a 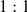 mapping from complex spinors to GA given in Eq. (9), so we will have a general three qubit state represented in GA as
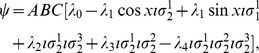
(48)which with the rotors gives us 15 degrees of freedom.

We desire though, a symmetrical three-qubit state in order to guarantee a fair game and so we construct
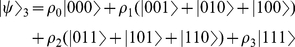
(49)as the most general symmetrical three qubit quantum state, with 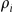 subject to the conventional normalization conditions. We might think to add complex phases to the four terms, however we find that this addition has no effect on the payoff or the NE and so can be neglected. This symmetrical state can be represented in GA, by referring to Eq. (11), as
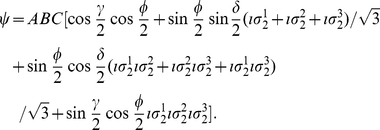
(50)If we set 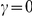 and 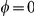 we find the product state 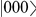, which we will constrain to return the classical game as for the GHZ state. For 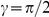 and 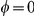 we produce the maximally entangled GHZ state and for 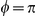 we have the W type states in a superposition controlled by 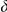. Using Eq. (50) and following the same calculation path used for the GHZ state, we can arrive at the NE, using the same condition for classical embedding as for the GHZ state, finding for Alice
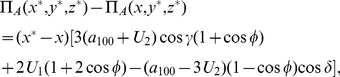
(51)where
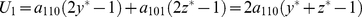
(52a)

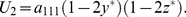
(52b)We can see the effect of the W type states in the 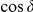 term and so it illustrates how both types of W states contribute. The reason they can both appear is because by demanding the classical embedding we have severely restricted the available unitary transformations available to transform the starting state.

#### The payoff relations

The payoff function for Alice given by
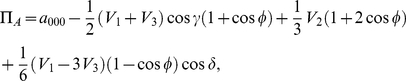
(53)where
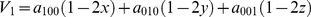
(54a)

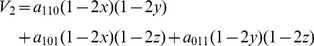
(54b)

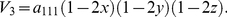
(54c)The payoff for Bob and Chris found by simply replacing 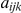 with 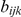 and 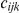 from their respective payoff matrices. When comparing with the payoff formula above with the classical result at 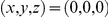, it is helpful to note that 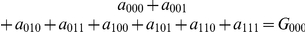 and generally 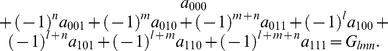


#### Uniform superposition state

If we select a uniform superposition state, with 
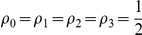
, that is, substituting 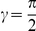, 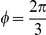 and 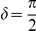, giving a product state H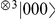, with H being the Hadamard operator, then we find that 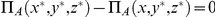 for Alice, and similarly for the other players. That is the payoff will be independent of the player choices and Eq. (53) gives 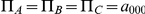. Where 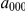 represents the average of all the entries in the payoff matrix, as expected for a uniform superposition state.

#### Prisoners' Dilemma

For the PD game from the previous section with the GHZ state, we found 
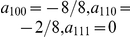
, so 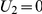, with the NE from Eq. (79) for the three players given by
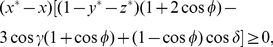
(55a)

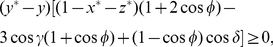
(55b)

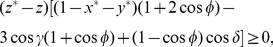
(55c)with the payoff for Alice given by
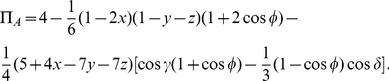
(56)We can see with 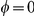 we recover the NE for the GHZ state, in Eq. (37).

#### Shifting of the NE compared to the GHZ state

We have the classical NE of 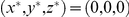 for 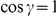 and 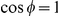, but we can see, that once again, we have a phase transition, as the entanglement increases, to a new NE of 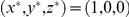, 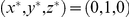 and 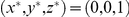.

The phase transition will be at 
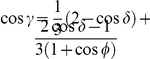
. We notice that as we increase the weighting towards the W state, by increasing 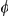, that it becomes easier to make the phase transition in comparison to the pure GHZ state, that is, we improve access to the phase transition as we introduce the weight of the 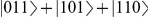 state. In fact, even at 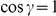, we can achieve the NE of 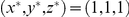, with 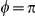, giving a payoff of 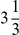 units.

#### Maximizing the payoff

Looking at the payoff function for Alice in Eq. (56), we can seek to maximize this function. The maximum achievable payoff is found to be 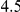, which is equal to the maximum payoff found for the GHZ state, see Fig. 2. Thus incorporating W type states into a superposition with the GHZ state, cannot improve the maximum payoff.

**Figure 3:**
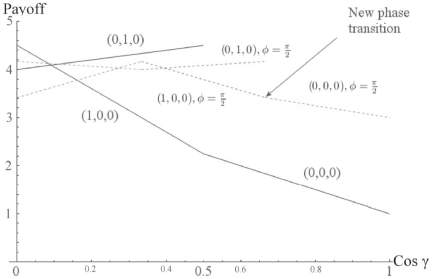
Phase transition in three-player quantum Prisoners' Dilemma with a general three qubit state. The solid lines indicate the phase transitions from Table 1, and shown in Fig. 1, with the dashed lines indicating the shifted transitions when the W-state is mixed in. We observe that new NE now arise at lower entanglement, at 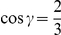, as indicated by the arrow pointer.

Observing Fig. 3, we can see that as we mix in the W state, that the phase transitions move to the right, with an extra offset available by changing 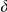, and the maximum payoff obtainable, will drop below the maximum achievable of 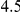 with the pure GHZ state. Fig. 3, shows the shifted NE from 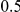 to 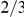 and payoffs for the case 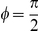 and 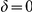.

## Discussion

A quantum version of a three-player two-strategy game is explored, where the player strategy sets remain classical but their payoffs are obtained from the outcome of quantum measurement performed, as in a typical EPR experiment. If players share a product state, then the quantum games reduces itself to the classical game, thus ensuring a faithful embedding of a mixed-strategy version of a classical three-player two-strategy game within the more general quantum version of the game.

For a general three-player two-strategy game, we find the governing equation for a strategy triplet forming a NE is given by Eq. (51) with the associated payoff relations obtained in Eq. (53). At zero entanglement the quantum game returns the same triplet(s) of NE as the classical mixed-strategy game and the payoff relations in the quantum game reduce to the trilinear form given in Eq. (35), equivalent to the classical game involving mixed-strategies. We find that even though the requirement to properly embed a classical game puts significant restrictions on the initial quantum states, we still have a degree of freedom, available with the entanglement angle 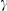, with which we can generate a new NE.

As a specific example the PD was found to have a NE of 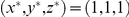 at high entanglement. For the GHZ state, the phase diagram is shown in Fig. 2, which is modulated with the inclusion of the W type states, by reducing the payoffs and sliding the NE closer to the classical region.

As our setup for a three-player quantum game involves players performing classical strategies, our conclusions are restricted by not only players sharing GHZ or W states but also by the EPR setting that we use. The most general form of the GHZ state permits a description in terms of a single entanglement parameter 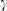. However, as the general W state involves three kets, the entanglement in such a state cannot be described by a single parameter. It appears that as for symmetric W states with equal superposition it is not possible to remove entanglement, therefore, embedding a classical game within the quantum game (while players share such states) is not possible in the EPR-type setup in which players can perform only classical strategies. Our results in this regard are general in that although they rely on the EPR setting, but not on a particular game as these use the parameters introduced in Eqs. (28a–28d) that can be evaluated for any game. Also, this is discussed in the Section 5, where games with general three-qubit symmetric states are considered, that include combination of GHZ and W states. However, the situation with sharing non-equally weighted superposition states can be entirely different, not considered in the present paper, but represents a useful extension for future work.

Our analysis shows that, with a quantization based on the EPR setting, a faithful embedding of a classical game can be achieved that also avoids an Enk-Pike type argument [32] because players' strategy sets are not extended relative to the classical game. However, with players sharing entangled states, while their strategy sets remain classical, our quantum games lead to new game-theoretic outcomes.

We also find that an analysis of three-player quantum games using Clifford's geometric algebra (GA) comes with some clear benefits, for instance, a better perception of the quantum mechanical situation involved and particularly an improved geometrical visualization of quantum mechanical operations. The same results using the familiar algebra with Pauli matrices may possibly be tractable but would certainly obscure intuition. Also, the simple expression given in (13) for the overlap probability between two quantum states in the 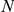-particle case is another benefit of the GA approach.

The results reported in the paper can be useful in a game-theoretic analysis of the EPR paradox. Bell's consideration of the EPR paradox usually implies the inconsistency between locality and completeness of quantum mechanics, or in more broader terms, simply the surprising nonlocal effects invoked by entanglement. However, one notices that these conclusions are merely sufficient but not necessary for the violation of Bell's inequality and that other interpretations are also reported [45], [51]–[54], especially, the interpretation based on the non-existence of a single probability space for incompatible experimental contexts [55]. This non-existence also presents a new route in constructing quantum games and the first step in this direction was taken in Ref [56]. Because such quantum games originate directly from the violation of Bell's inequality, they allow a discussion of the EPR paradox in the context of game theory. This is also supported by the fact that for quantum games with players sharing entanglement, a game-theoretic analysis that involves Bell's settings [26]–[28] has been reported in Refs [57], [58].

A variety of other classical games could now be adapted and applied to this three-player framework, with new NE being expected. The present study of three-player quantum games can also be naturally extended to analyze the N-player quantum games. We believe that the mathematical formalism of GA permits this in a way not possible using the usual complex matrices. Also, this extension could be fruitfully exploited in developing a game-theoretic perspective on quantum search algorithms and quantum walks. We find that our analysis can be helpful in providing an alternative viewpoint (with emphasis on underlying geometry) on multi-party entanglement shared by a group of individuals (players), while they have conflicting interests and can perform only classical actions on the quantum state. That is, a viewpoint that is motivated by the geometrical perspective that Clifford's geometric algebra provides. Such situations take place in the area of quantum communication and particularly in quantum cryptography [59]–[61].

## Supporting Information

Appendix S1 (PDF)
