# Supplementary material for: Analyzing Three-Player Quantum Games in an EPR Type Setup
Source: PLoS One. 2011 Jul 27;6(7):e21623. doi: 10.1371/journal.pone.0021623 (PMC3144879; doi:10.1371/journal.pone.0021623)
Supplement: Appendix S1 — (PDF) [file pone.0021623.s001.pdf]

Appendix

Calculating the observables

The following three results are useful when calculating the observables for a given measurement direction κ. If we have a rotor defined as

$$R = \mathrm{e}^{-\alpha_3 \iota\sigma_3/2}\mathrm{e}^{-\alpha_1 \iota\sigma_2/2}\mathrm{e}^{-\alpha_2 \iota\sigma_3/2}, \tag{1}$$

then we find

$$\begin{aligned} R\iota\sigma_3 R^\dagger &= \iota \mathrm{e}^{-\alpha_3 \iota\sigma_3/2}\mathrm{e}^{-\alpha_1 \iota\sigma_2/2}\mathrm{e}^{-\alpha_2 \iota\sigma_3/2}\sigma_3 \mathrm{e}^{\alpha_2 \iota\sigma_3/2}\mathrm{e}^{\alpha_1 \iota\sigma_2/2}\mathrm{e}^{\alpha_3 \iota\sigma_3/2} \\ &= \iota\sigma_3 \mathrm{e}^{-\alpha_3 \iota\sigma_3/2}\mathrm{e}^{\alpha_1 \iota\sigma_2}\mathrm{e}^{\alpha_3 \iota\sigma_3/2} \\ &= \iota\sigma_3 \cos\alpha_1 + \iota\sigma_1 \sin\alpha_1 \mathrm{e}^{\alpha_3 \iota\sigma_3} \\ &= \cos\alpha_1 \iota\sigma_3 + \sin\alpha_1 \cos\alpha_3 \iota\sigma_1 + \sin\alpha_1 \sin\alpha_3 \iota\sigma_2. \end{aligned} \tag{2}$$

Therefore acting with the measurement operator $\iota\sigma_3 \mathrm{e}^{\kappa\iota\sigma_2}$, we find

$$\begin{aligned} \langle R\iota\sigma_3 R^\dagger \iota\sigma_3 \mathrm{e}^{\kappa\iota\sigma_2}\rangle_0 &= (\cos\alpha_1 \iota\sigma_3 + \sin\alpha_1 \cos\alpha_3 \iota\sigma_1 + \sin\alpha_1 \sin\alpha_3 \iota\sigma_2)\iota\sigma_3(\cos\kappa + \iota\sigma_2 \sin\kappa) \\ &= -\cos\alpha_1 \cos\kappa - \cos\alpha_3 \sin\alpha_1 \sin\kappa = -X(\kappa). \end{aligned} \tag{3}$$

Similarly we have for the other two cases

$$\langle \iota R\sigma_2 R^\dagger \iota\sigma_3 \mathrm{e}^{\kappa\iota\sigma_2}\rangle_0 = \sin\kappa(\cos\alpha_2 \sin\alpha_3 + \sin\alpha_2 \cos\alpha_3 \cos\alpha_1) - \sin\alpha_1 \sin\alpha_2 \cos\kappa, \tag{4a}$$

$$\langle \iota R\sigma_1 R^\dagger \iota\sigma_3 \mathrm{e}^{\kappa\iota\sigma_2}\rangle_0 = -\sin\kappa(\cos\alpha_1 \cos\alpha_2 \cos\alpha_3 - \sin\alpha_2 \sin\alpha_3) + \sin\alpha_1 \cos\alpha_2 \cos\kappa. \tag{4b}$$
